# Comparative Phenolic Profiling and Biological Evaluation of Original Wine, Processed Dealcoholized Wine, and Fully Evaporated Dry Wine Extract

**DOI:** 10.3390/molecules31213843

**Published:** 2026-10-24

**Authors:** Katarina G. Marković, Ivana D. Radojević, Katarina Ćirković, Andrija Ćirić, Stefan Marković, Edina Avdović, Dušica Simijonović, Nenad M. Zlatić, Sanja Lj. Matić, Mirjana Ž. Grujović

**Affiliations:** 1 Institute for Information Technologies, Department of Science, University of Kragujevac, Liceja Kneževine Srbije 1a, 34000 Kragujevac, Serbia; katarina.mladenovic@pmf.kg.ac.rs (K.G.M.); edina.avdovic@pmf.kg.ac.rs (E.A.); dusica.simijonovic@uni.kg.ac.rs (D.S.); sanjamatic@kg.ac.rs (S.L.M.); 2 Faculty of Science, Department of Biology and Ecology, University of Kragujevac, Radoja Domanovića 12, 34000 Kragujevac, Serbia; ivana.radojevic@pmf.kg.ac.rs (I.D.R.); katarina.cirkovic@pmf.kg.ac.rs (K.Ć.); stefan.markovic@pmf.kg.ac.rs (S.M.); nenad.zlatic@pmf.kg.ac.rs (N.M.Z.); 3 Faculty of Science, Department of Chemistry, University of Kragujevac, Radoja Domanovića 12, 34000 Kragujevac, Serbia; andrija.ciric@pmf.kg.ac.rs

**Keywords:** dealcoholized wine, phenolic compounds, HPLC, antioxidant activity, antimicrobial activity, antibiofilm activity, oxidative damage, anthocyanins, flavonoids, functional beverages

## Abstract

The growing demand for low- and non-alcoholic beverages has stimulated interest in the bioactive properties of dealcoholized wine. This comparative pilot study characterized three sample types derived from the same red wine: original wine (OW), processed dealcoholized wine (PDW), and fully evaporated dry wine extract (FDE). Phenolic composition was determined spectrophotometrically and by HPLC-DAD, and antioxidant (DPPH, ABTS), antimicrobial, antibiofilm, and in vitro DNA-protective activities were evaluated. Compared with OW, PDW showed higher total phenolic (831.91 vs. 773.56 mg GAE/L), flavonoid (82.49 vs. 72.84 mg RUE/L), tannin (70.73 vs. 60.58 mg/L), and epicatechin (159.59 vs. 52.38 mg/L) concentrations; however, because 500 mL of wine was reduced to approximately 100 mL, mass balances indicated that PDW contained only 22–30% of these constituents. FDE contained approximately 68% of the anthocyanins of the starting wine (19.02 mg/L wine equivalent). OW and PDW showed comparable antioxidant (0.98 and 1.17 mmol TE/L, DPPH) and antimicrobial activities, with *Staphylococcus aureus* being the most susceptible species (MIC ≤ 0.391 µL/mL). Only FDE inhibited biofilm formation, with MBIC values of 2.5–10 mg/mL after 24 h. All samples reduced hydroxyl- and peroxyl radical-induced DNA damage. These findings characterize the investigated samples and provide a basis for controlled studies of wine processing. Dealcoholized wine and dry wine extracts may represent potential starting materials for low-alcohol functional beverages or wine-derived food ingredients, although processing conditions that limit phenolic losses need to be optimized and their biological relevance confirmed in further studies.

## 1. Introduction

Wine is a complex matrix rich in biologically active compounds, among which phenolic constituents represent one of the most important groups contributing to its functional properties [1]. These compounds, originating primarily from grape skins, seeds, and stems, include phenolic acids, flavonoids, and tannins, and are known to play a crucial role in determining the sensory characteristics, stability, and health-related effects of wine [1,2,3,4]. In recent years, increasing attention has been directed toward the investigation of wine phenolics due to their pronounced antioxidant, antimicrobial, and antibiofilm activities, which are of significant interest in food science, pharmacology, and biomedical research [5,6,7].

The antioxidant capacity of wine is largely attributed to its high content of phenolic compounds, particularly total phenols, flavonoids, and proanthocyanidins, which act as effective free radical scavengers and metal chelators [1,2,6,7,8,9]. Flavonoids such as quercetin, rutin, and catechins, as well as phenolic acids including gallic and caffeic acids, are especially recognized for their strong antioxidant potential [10]. Proanthocyanidins, as condensed tannins, further contribute to antioxidant activity through their ability to donate hydrogen atoms and stabilize free radicals [11]. Consequently, the determination of total phenolic content (TPC), total flavonoid content (TFC), and total proanthocyanidin content (TPAC), together with antioxidant assays, provides essential insight into the bioactive potential of wine [2].

In addition to antioxidant properties, wine phenolics have been extensively studied for their antimicrobial activity against a wide range of microorganisms [1,2,6,7,12]. These compounds can disrupt microbial cell membranes, interfere with enzyme activity, and induce oxidative stress, ultimately leading to growth inhibition or cell death [12,13,14]. Importantly, phenolics have also been shown to affect biofilm formation, a critical virulence factor that enhances microbial resistance to environmental stress and antimicrobial agents [15]. The ability of phenolic compounds to inhibit quorum sensing, reduce extracellular polymeric substance (EPS) production, and prevent microbial adhesion makes them promising candidates for controlling biofilm-associated infections [15,16,17,18]. Moreover, clinical and in vitro studies have indicated that wine and wine-derived phenolics may protect DNA against oxidative strand damage induced by reactive oxygen species [19,20].

The composition and concentration of phenolic compounds in wine can be significantly influenced by vinification processes, including fermentation conditions and post-fermentation treatments such as dealcoholization [21]. Partial or complete removal of ethanol may alter the extraction, stability, and availability of phenolic constituents, thereby affecting the biological properties of the final product [22]. In particular, concentration of non-volatile wine constituents through solvent removal may modify the relative abundance of bioactive compounds and consequently affect antimicrobial and antibiofilm efficacy [23].

Therefore, the aim of this comparative pilot study was to characterize and compare the chemical composition and biological activities of three sample types derived from the same red wine: original wine (OW), processed dealcoholized wine (PDW), and fully evaporated dry wine extract (FDE). Red wine was selected as the model matrix because of its complex and phenolic-rich composition, encompassing phenolic acids, flavonoids, tannins, and anthocyanins, which enables the comparative evaluation of a broad range of phenolic constituents. Special emphasis was placed on phenolic profiling by HPLC at multiple wavelengths, together with the determination of total phenolic, flavonoid, tannin, and anthocyanin contents and their relationships with antioxidant capacity. The antimicrobial and antibiofilm activities of the three sample types were also investigated, alongside their in vitro DNA-protective capacity against hydroxyl- and peroxyl radical-induced oxidative damage. This integrated approach provides an initial comparative characterization of the chemical and biological properties of these three wine-derived sample types and a basis for future controlled investigations of processing-related changes.

## 2. Results

### 2.1. Phenolic Composition of Wine Samples

The phenolic composition of wine samples was evaluated through the determination of total phenolic, flavonoid, tannin, and anthocyanin contents (Table 1), complemented by HPLC analysis (Table 2) for the identification and quantification of individual polyphenolic compounds. This comprehensive approach enabled a detailed characterization of the phenolic profile and provided insight into the contribution of specific bioactive constituents to the overall chemical properties of the investigated samples.

The measured TPC, TFC, TTC, and TAC values were higher in PDW than in OW (Table 1). For FDE, the experimentally determined dry-extract yield of 27.17 g/L of starting wine enabled the values originally expressed on a dry-mass basis (mg/g) to be additionally converted to wine-volume equivalents. On this basis, FDE corresponded to 1074.85 ± 16.85 mg GAE/L for TPC, 213.28 ± 5.43 mg RUE/L for TFC, 67.11 ± 0.27 mg/L for TTC and 19.02 ± 0.91 mg/L for TAC. These wine-volume-equivalent values are provided to facilitate descriptive comparison among the three sample types. To assess retention or loss of these constituents, mass balances were calculated relative to the 500 mL of OW used for the preparation of PDW and FDE (Table 1). Because PDW was obtained by reducing 500 mL of OW to approximately 100 mL, its higher measured concentrations corresponded to only approximately 22% (TPC), 23% (TFC), 23% (TTC), and 30% (TAC) of the amounts present in the starting wine. For FDE, the calculated recoveries were 139% (TPC), 293% (TFC), 111% (TTC), and 68% (TAC). Recoveries exceeding 100% are unlikely to reflect a true increase in total phenolic content and more likely indicate that the Folin–Ciocalteu, AlCl_3_, and tannin assays responded differently to the liquid wine and the reconstituted dry extract; these values were therefore not interpreted as evidence of phenolic enrichment. FDE showed higher TPC and TFC values than OW and PDW, a TTC value within a similar range to those of the liquid samples, and a lower TAC value than both liquid samples.

HPLC analysis performed at 254, 280, 360, and 520 nm (Supplementary Figures S1–S4) revealed differences in individual phenolic constituents among the three investigated sample types. The chromatographic profiles of PDW and OW were broadly similar, although quantitative differences were observed for several individual compounds. Compared with OW, PDW contained higher concentrations of chlorogenic, caffeic, p-coumaric, and ferulic acids, as well as epicatechin, rutin, quercetin, and apigenin, whereas lower concentrations were observed for gallic, syringic, and sinapic acids, 3,4- and 3,5-dihydroxybenzoic acids, myricetin, and naringenin. Epicatechin showed the most pronounced difference, with concentrations of 52.38 ± 1.03 mg/L in OW and 159.59 ± 0.20 mg/L in PDW.

All quantified phenolic compounds were also detected in FDE. To facilitate comparison with the liquid samples, FDE concentrations originally determined on a dry-mass basis (mg/g) were additionally converted to wine-volume equivalents (mg/L of starting wine) using the experimentally determined dry-extract yield of 27.17 g/L. The normalized data revealed compound-specific patterns rather than a uniform reduction in phenolic constituents. For example, the wine-equivalent concentrations of epicatechin, chlorogenic acid, sinapic acid, and apigenin in FDE were 138.57 ± 1.63, 34.78 ± 0.27, 31.79 ± 1.36, and 15.22 ± 0.27 mg/L, respectively. In contrast, several compounds, including gallic, caffeic, syringic, ferulic acids, rutin, naringin, myricetin, and naringenin, were present at lower wine-equivalent concentrations in FDE than in one or both liquid samples. These findings indicate distinct quantitative distributions of individual phenolic compounds among OW, PDW, and FDE rather than a uniform pattern across phenolic classes. Mass-balance calculations based on the summed concentrations of the 17 quantified compounds indicated that PDW contained approximately 26% of the amount present in the starting wine, consistent with the spectrophotometric data, whereas the corresponding value for FDE was approximately 115%. In FDE, recoveries exceeding 100% were obtained for several individual compounds, including apigenin (668%), chrysin (529%), 3,4-dihydroxybenzoic acid (503%), quercetin (395%), epicatechin (265%), and chlorogenic acid (170%), whereas lower recoveries were obtained for rutin (53%), syringic acid (47%), naringin (44%), and myricetin (26%).

### 2.2. Antioxidant Activity of Wine Samples

Antioxidant potential was evaluated based on the ability of wine samples to neutralize DPPH and ABTS radicals. The results are presented in Table 3 as Trolox equivalents (TE = mmol TE/L).

OW and PDW showed comparable TE values in both assays (0.98 and 1.17 mmol TE/L in the DPPH assay and 4.77 and 4.62 mmol TE/L in the ABTS assay, respectively). For FDE, TE values of 9.33 mmol TE/L (DPPH) and 49.37 mmol TE/L (ABTS) were obtained for the reconstituted extract. Because FDE was tested as a reconstituted dry extract rather than as a liquid wine, these values are not directly comparable with those of OW and PDW, and no ranking of antioxidant activity among the three sample types is made.

Across all samples, antioxidant activity was generally more pronounced in the ABTS assay than in the DPPH assay. In particular, TE values obtained in the ABTS assay were approximately fivefold higher than those observed in the DPPH assay. This disparity likely reflects differences in the reactivity of the radical species employed and the relative efficiency of the antioxidant constituents depending on the underlying radical system.

### 2.3. Antimicrobial Activity of Wine Samples

The antimicrobial activity of OW, PDW, and FDE varied according to the tested microorganism and sample type (Table 4). Because OW and PDW were tested on a volume basis (µL/mL), whereas FDE was tested on a mass basis (mg/mL), the original values are reported in their experimentally determined units. To provide a common quantitative basis for descriptive comparison, FDE values were additionally converted to starting-wine-volume equivalents using the experimentally determined dry-extract yield of 27.17 g/L.

Among Gram-positive bacteria, *Staphylococcus aureus* strains showed the greatest susceptibility to the investigated samples. OW and PDW showed MIC values as low as 0.391 µL/mL and <0.391 µL/mL, respectively, whereas the FDE MIC was <0.156 mg/mL, corresponding to <5.74 µL/mL on a starting-wine-volume-equivalent basis. *Bacillus* spp. showed lower susceptibility to OW and PDW, with MIC values of 25 or >25 µL/mL, whereas FDE MIC values ranged from 2.5 to 5 mg/mL, corresponding to 92.01–184.03 µL/mL on a starting-wine-volume-equivalent basis. *Lactiplantibacillus plantarum* was not inhibited by OW or PDW at the highest tested concentration, whereas an MIC of 5 mg/mL (184.03 µL/mL starting-wine equivalent) was determined for FDE.

Gram-negative bacteria, including *Escherichia coli*, *Pseudomonas aeruginosa*, and *Proteus mirabilis*, generally showed limited susceptibility under the tested conditions. OW and PDW had MIC values of 25 or >25 µL/mL, while FDE MIC values were 5 or >5 mg/mL for most of these strains, corresponding to 184.03 or >184.03 µL/mL on a starting-wine-volume-equivalent basis.

The investigated samples also exhibited limited antifungal activity. *Candida albicans* was the only fungal species for which defined MIC values were obtained for all three samples, with MIC values of 25 µL/mL for OW and PDW and 5 mg/mL for FDE, corresponding to 184.03 µL/mL on a starting-wine-volume-equivalent basis. In contrast, *Saccharomyces boulardii* and the tested filamentous fungi were not inhibited at the highest concentrations tested. Overall, the antimicrobial activity was strongly microorganism-dependent. Yield-based normalization of FDE provides a more appropriate quantitative basis for descriptive comparison with the liquid samples and demonstrates that lower numerical MIC values expressed in mg/mL should not be interpreted directly as greater antimicrobial activity than values expressed in µL/mL. This normalization reflects the amount of starting wine required to obtain a given mass of FDE and does not imply physicochemical or biological equivalence between FDE and the corresponding volume of liquid wine.

### 2.4. Antibiofilm Activity of Wine Samples

The antibiofilm activity of OW, PDW, and FDE against selected Gram-positive bacteria is presented in Table 5. Overall, OW and PDW exhibited no detectable inhibition of biofilm formation, with MBIC values exceeding 25 µL/mL for all tested strains after both 24 h and 48 h of incubation.

FDE showed measurable antibiofilm activity against all four tested bacterial strains, with MBIC values ranging from 2.5 to 10 mg/mL after 24 h, corresponding to 92.01–368.05 µL/mL on a starting-wine-volume-equivalent basis. After 48 h, MBIC values were 10 mg/mL for both *S. aureus* strains and *B. cereus*, whereas a value >10 mg/mL was observed for *B. subtilis* ATCC 6633. The most pronounced time-dependent change was observed for *B. cereus*, for which the MBIC increased from 2.5 mg/mL (92.01 µL/mL starting-wine equivalent) at 24 h to 10 mg/mL (368.05 µL/mL) at 48 h, indicating lower antibiofilm efficacy with prolonged incubation. No biofilm formation was detected for *B. subtilis* IP 5832; therefore, antibiofilm activity could not be evaluated for this strain. Tetracycline, included as positive control, showed strain-dependent MBIC values after 48 h, as presented in Table 5.

### 2.5. DNA-Protective Capacity of Wine Samples

To investigate the in vitro DNA-protective capacity of wine samples, oxidative damage was generated using hydrogen peroxide (H_2_O_2_) and 2,2′-azobis(2-amidinopropane) dihydrochloride (AAPH), and the resulting effects were evaluated by agarose gel electrophoresis. The extent of DNA protection was determined by comparing band integrity and fluorescence intensity between control samples and samples treated with wine at amounts of 3.125, 6.25, 12.5, 25, and 50 μg of each sample, corresponding to approximately 3.1–50 nL of wine for OW and PDW and to 3.125–50 μg of dry extract for FDE. Because the doses of the liquid samples and of FDE are not directly comparable on a quantitative basis (Section 4.6.1), the DNA-protective effects were evaluated for each sample separately.

**Figure 1:**
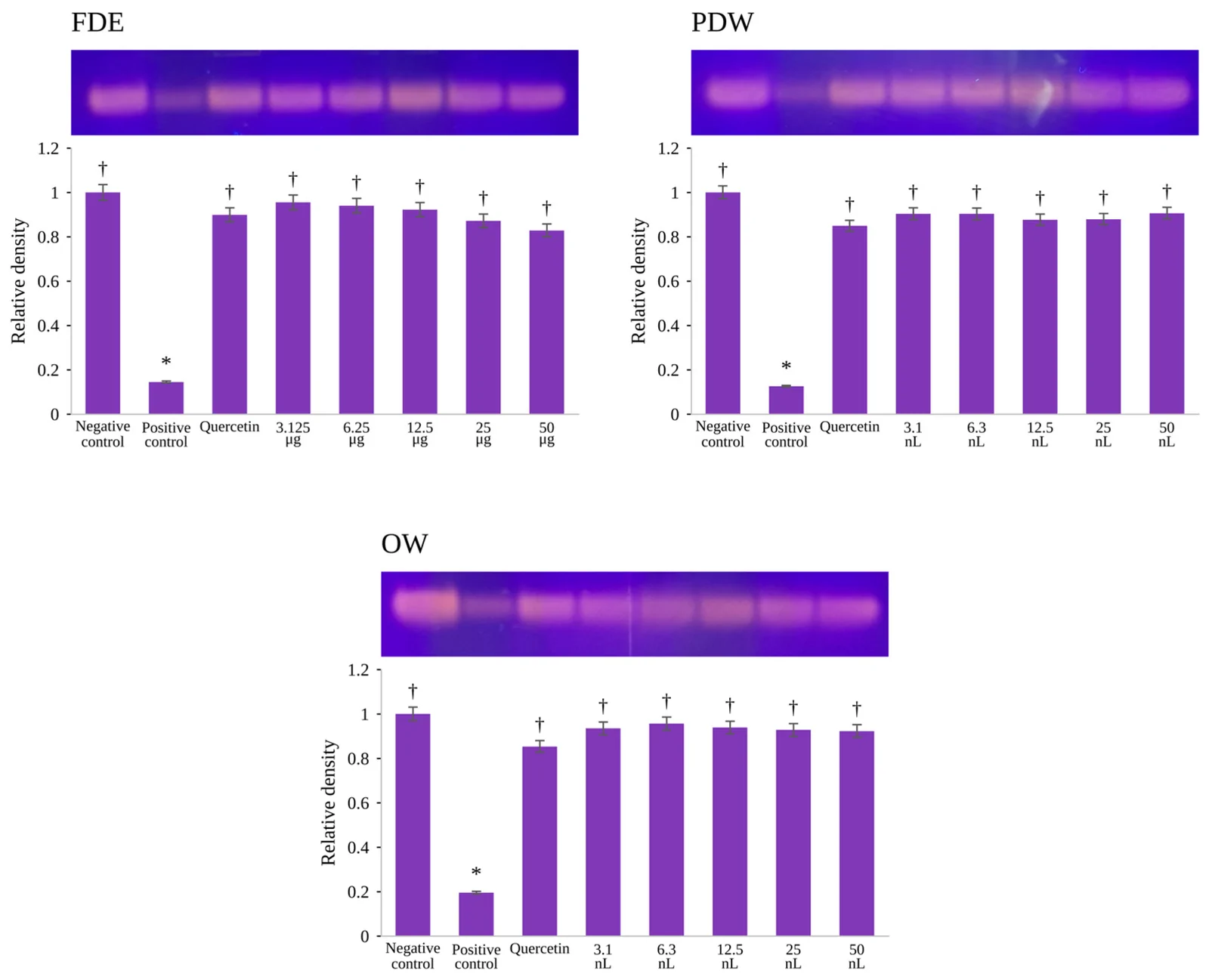
Protective activity of wine samples against hydroxyl radical-mediated DNA damage. DNA from salmon sperm (lane 1, negative control); DNA damage control (lane 2, positive control); quercetin (lane 3, standard); FDE, PDW, and OW (panels labelled accordingly) at 3.1, 6.3, 12.5, 25, and 50 nL of OW and PDW (corresponding to approximately 3.125–50 μg of wine) or 3.125, 6.25, 12.5, 25, and 50 μg of FDE dry extract (lanes 4, 5, 6, 7, and 8); * *p* < 0.05 when compared with the negative control group; † *p* < 0.05 when compared with the positive control group.

**Figure 2:**
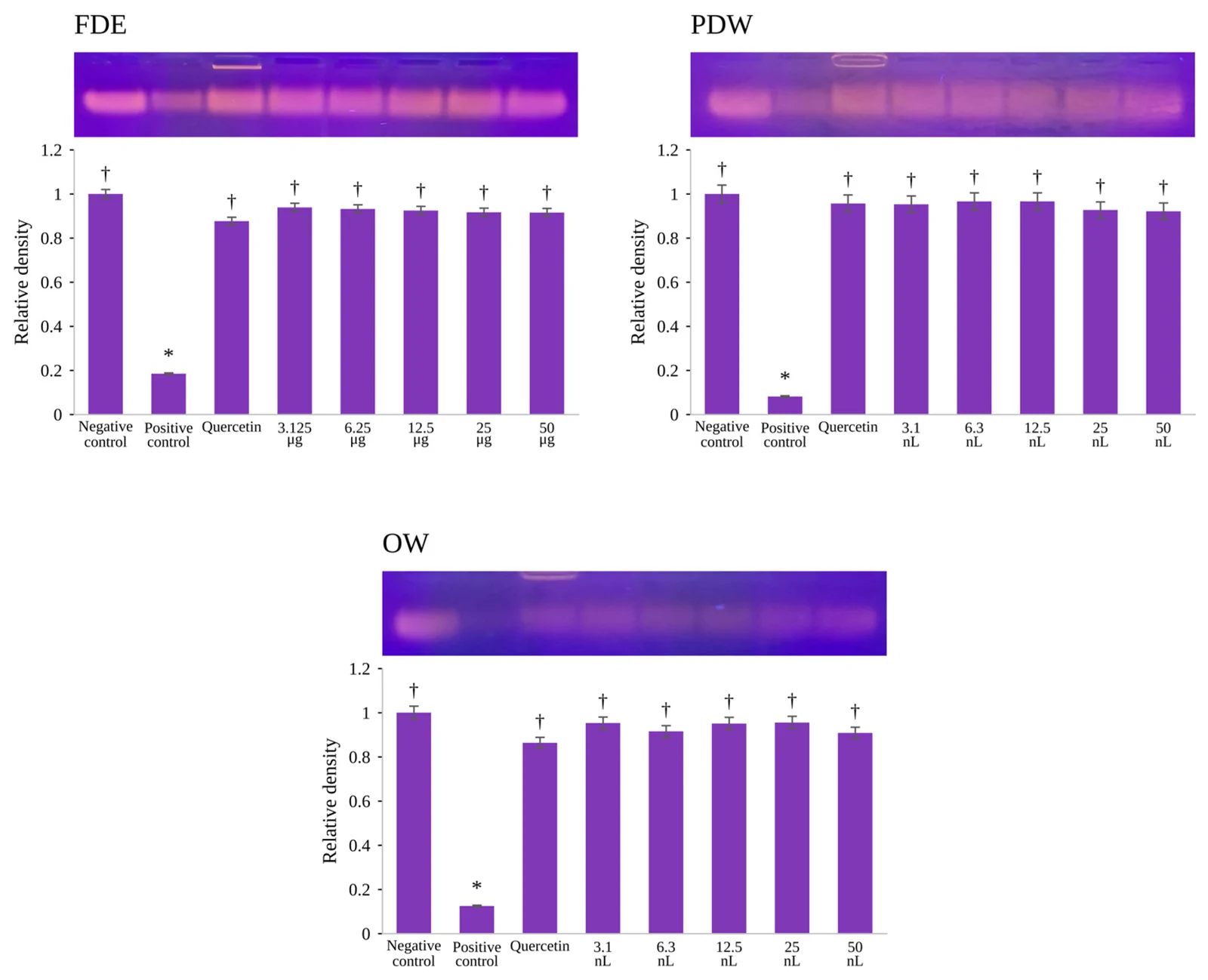
Protective activity of wine samples against peroxyl radical-mediated DNA damage. DNA from salmon sperm (lane 1, negative control); DNA damage control (lane 2, positive control); quercetin (lane 3, standard); FDE, PDW, and OW (panels labelled accordingly) at 3.1, 6.3, 12.5, 25, and 50 nL of OW and PDW (corresponding to approximately 3.125–50 μg of wine) or 3.125, 6.25, 12.5, 25, and 50 μg of FDE dry extract (lanes 4, 5, 6, 7, and 8); * *p* < 0.05 when compared with the negative control group; † *p* < 0.05 when compared with the positive control group.

Exposure of DNA to the FeSO_4_/H_2_O_2_ system or the AAPH system induced pronounced oxidative damage in the positive control, as evidenced by extensive band smearing and a marked reduction in fluorescence intensity on agarose gels (Figure 1 and Figure 2, lane 2). In contrast, the negative control exhibited intact, compact DNA bands with preserved fluorescence, supporting the absence of oxidative degradation (Figure 1 and Figure 2, lane 1).

In the hydroxyl radical model generated via the Fenton reaction, all tested wine samples (OW, PDW and FDE) demonstrated protective effects against DNA strand damage across the investigated dose range, without a clear dose-dependent trend (Figure 1). Compared to the positive control, which showed extensive DNA degradation, treatment with wine samples resulted in the preservation of DNA integrity, reflected by more intense and well-defined bands on agarose gels.

A similar protective pattern was observed in the peroxyl radical-induced model initiated by AAPH (Figure 2). All wine samples significantly attenuated oxidative DNA damage across all tested concentrations, as indicated by improved band integrity and fluorescence intensity relative to the positive control. As in the hydroxyl radical system, all samples consistently demonstrated DNA-protective activity, without a clear dose-dependent trend (Figure 2).

## 3. Discussion

The present comparative pilot study characterized the phenolic composition and biological properties of three sample types derived from the same red wine: OW, PDW, and FDE. The observed differences are therefore interpreted primarily as characteristics of the investigated samples rather than as definitive causal effects of the corresponding processing procedures. Physicochemical characterization of the samples (Section 4.1.1) showed that PDW preparation reduced the ethanol content from 12.0% to 0.2% (*v*/*v*) and free and total SO_2_ from 2.1 and 5.2 mg/L, respectively, to below 1 mg/L. The low SO_2_ content of OW is consistent with the absence of SO_2_ addition during winemaking and is well below the limits prescribed by current European regulations [24]. Reductions in free and total SO_2_ have also been reported following wine dealcoholization, although their extent depends on the applied technique, processing conditions, and wine matrix [25,26]. Because total SO_2_ comprises both free and bound fractions [27], the absence of detectable total SO_2_ in PDW indicates that bound SO_2_ forms were also largely removed or dissociated during processing. The pH changed from 3.54 in OW to 3.39 in PDW, whereas the reconstituted FDE had a pH of 3.52. The latter value should be interpreted cautiously because FDE was measured after reconstitution at a defined concentration (30 mg/mL) and is therefore not directly equivalent to the pH measured in the liquid wine samples.

PDW exhibited higher measured TPC, TFC, TTC, and TAC values than OW and showed a broadly similar chromatographic fingerprint, although substantial quantitative differences were observed for several individual phenolic compounds. Changes in the physicochemical characteristics of the wine matrix, including the marked reduction in ethanol and SO_2_, may also have affected the measured phenolic profile; however, the relative contributions of these factors cannot be distinguished within the present experimental design. Previous studies have similarly shown that changes in phenolic composition during wine dealcoholization are strongly dependent on the processing technique and conditions, with thermal methods in particular potentially increasing the apparent concentration of retained non-volatile constituents through concentration effects [28,29]. Importantly, the preparation of PDW reduced the sample volume from 500 mL to approximately 100 mL (approximately five-fold). Mass-balance calculations showed that, despite the higher measured concentrations, PDW contained only approximately 22%, 23%, 23%, and 30% of the TPC, TFC, TTC, and TAC present in the starting wine, respectively. Therefore, the higher measured concentrations in PDW reflect volume reduction rather than retention or enrichment of these constituents, and they occurred despite a substantial loss during processing, which may be related to thermal exposure, oxidation, precipitation, or other matrix-related changes. Consistent with this, a visible precipitate formed during PDW preparation and was not included in the analysed sample. The approximately five-fold concentration of the wine during evaporation would be expected to exceed the solubility of potassium hydrogen tartrate and to promote the precipitation of polymeric pigments and tannin–protein or tannin–polysaccharide complexes [30,31], and co-precipitation or adsorption of phenolic compounds onto this material is likely to have contributed substantially to their lower recovery in PDW. In contrast, no fraction was separated during FDE preparation, so any precipitated material remained in the dry residue.

The experimentally determined FDE yield also enabled the dry-mass-based results to be expressed as wine-volume equivalents, providing a more appropriate basis for comparison with OW and PDW. When normalized to the starting wine volume, FDE showed higher TPC and TFC values than the liquid samples, whereas TTC remained within a similar range and TAC was lower (19.02 mg/L, corresponding to approximately 68% of the anthocyanins present in the starting wine). These results differ from the impression obtained when FDE is considered only on a dry-mass basis and demonstrate the importance of accounting for extract yield when comparing liquid wine with a dry wine extract. Nevertheless, these spectrophotometrically derived wine-equivalent values should be interpreted cautiously because differences in matrix composition and analytical response between liquid wine and reconstituted dry extract may influence the measured values. In particular, the calculated FDE recoveries exceeding 100% for TPC (139%), TFC (293%), and TTC (111%) are unlikely to represent true recoveries and more likely indicate that these assays responded differently to the two matrices [32] and were therefore not interpreted as evidence of phenolic enrichment. One possible contributor is the release of phenolic compounds bound in colloidal structures, polymeric pigments or other complexes in the wine matrix [33], which may become more accessible to the assay reagents after evaporation to dryness and reconstitution of the extract in methanol.

The HPLC data further demonstrated that the differences among the samples were compound-specific rather than reflecting a uniform loss or enrichment of phenolic constituents. PDW contained markedly more epicatechin than OW (159.59 vs. 52.38 mg/L), together with higher concentrations of several other compounds, including caffeic and ferulic acids, rutin, quercetin, and apigenin. After normalization of FDE concentrations to the starting wine volume based on the experimentally determined extract yield, several compounds remained present at substantial wine-volume-equivalent levels. Notably, epicatechin corresponded to 138.57 mg/L of starting-wine equivalent, approaching the concentration measured in PDW, while chlorogenic acid and apigenin corresponded to 34.78 and 15.22 mg/L, respectively. Conversely, lower wine-volume-equivalent concentrations were obtained for several other phenolics, including gallic and syringic acids, rutin, naringin, myricetin, and naringenin. Collectively, these findings demonstrate distinct compound-specific phenolic profiles among OW, PDW, and FDE rather than a uniform pattern of phenolic depletion or enrichment. Such compound-dependent responses are consistent with previous reports showing that changes in the phenolic composition of dealcoholized red wines vary according to the individual phenolic class or compound and the processing conditions applied [28]. HPLC-based mass balances showed that, in PDW, the summed individual phenolics corresponded to approximately 26% of those in the starting wine, in agreement with the spectrophotometric recoveries. In FDE, however, several compounds were recovered in amounts exceeding those present in the starting wine. Because HPLC quantification is not subject to the non-specific interferences of the Folin–Ciocalteu and AlCl_3_ assays, these values suggest that some compounds may have been formed during evaporation to dryness. The pattern of these changes is chemically consistent: recoveries above 100% were observed mainly for flavonoid aglycones (apigenin, chrysin, and quercetin), whereas the corresponding glycosides were recovered at lower levels (rutin, 53%; naringin, 44%), which is compatible with hydrolysis of glycosides in the progressively acidified matrix during evaporation to dryness. In addition, protocatechuic (3,4-dihydroxybenzoic) acid is a known degradation product of anthocyanins, consistent with the partial loss of anthocyanins in FDE (TAC recovery of 68%), and epicatechin may be released by cleavage of proanthocyanidins. However, these mechanisms were not experimentally verified, and the recoveries of compounds present at very low concentrations in the analyzed solutions, such as quercetin and chrysin, are subject to considerable analytical uncertainty and should be interpreted with caution. It should also be noted that the largest relative increases corresponded to small absolute differences: chrysin and quercetin were present in OW at only 0.41 and 0.55 mg/L, and their wine-equivalent concentrations in FDE exceeded those in OW by less than 2 mg/L, whereas epicatechin was the only compound for which the absolute difference was substantial. Release of phenolics bound in the wine matrix upon reconstitution of the dry extract may also have contributed to the higher HPLC recoveries. Differences in phenolic composition among the investigated samples were accompanied by differences in their antioxidant properties. Although PDW had higher measured total phenolic, flavonoid, and tannin concentrations than OW, both samples exhibited comparable radical-scavenging activity, suggesting that antioxidant capacity depends not only on the total phenolic content but also on the qualitative composition and synergistic interactions among individual phenolic compounds. Among the quantified constituents, the approximately three-fold higher epicatechin concentration in PDW is particularly noteworthy, as flavan-3-ols are recognized as major contributors to the antioxidant capacity of red wines through their ability to donate hydrogen atoms or electrons and neutralize reactive oxygen species [8,34,35]. In addition to their antioxidant properties, these compounds have also been associated with anti-inflammatory, cardioprotective, and antimicrobial activities, further enhancing the functional value of red wine [8,36].

The observed differences in antioxidant activity between the DPPH and ABTS assays may be attributed to differences in the reactivity of the bioactive compounds toward the respective radicals. Whereas the DPPH assay primarily evaluates hydrogen atom transfer reactions, the ABTS assay is based on both hydrogen atom and electron transfer mechanisms, allowing the detection of antioxidant activity in a broader range of hydrophilic and lipophilic compounds [37]. Consequently, individual phenolics may exhibit different responses depending on their chemical structure and redox properties [38]. The consistently higher Trolox equivalent values obtained with the ABTS assay, particularly for FDE, may reflect the contribution of antioxidant compounds with diverse polarities and reactivities; therefore, the combined use of DPPH and ABTS provides a more comprehensive assessment of antioxidant capacity.

The antimicrobial activity observed in the present study is generally consistent with previously published data on the antimicrobial properties of wine and wine-derived extracts. Čomić et al. [2] similarly reported antimicrobial activity of red wine extracts obtained after evaporation to dryness and discussed the possible contribution of non-volatile bioactive constituents to this activity. In the present study, antimicrobial susceptibility was strongly species- and strain-dependent, with the two *S. aureus* strains showing the greatest susceptibility among the tested bacteria. In contrast, most Gram-negative bacteria exhibited low susceptibility under the applied conditions, although a simple Gram-positive/Gram-negative distinction does not fully explain the observed pattern. Previous studies have reported greater susceptibility of Gram-positive bacteria to grape-derived phenolic compounds, which has been associated with structural differences in the bacterial cell envelope and the restrictive role of the outer membrane of Gram-negative bacteria [39]. The pronounced activity against *S. aureus* observed in the present study is also consistent with Møretrø and Daeschel [40], who reported inhibitory activity of wine against this species at relatively high wine concentrations (MIC ≈ 35% (*v*/*v*)). Direct quantitative comparison with previous studies should, however, be made cautiously because of differences in sample preparation, concentration units, wine composition, and experimental conditions. OW and PDW exhibited broadly comparable antimicrobial activity against most tested microorganisms. However, because ethanol-, SO_2_-, and matrix-matched controls were not included, the individual contributions of ethanol, SO_2_, phenolic compounds, and other matrix constituents to the observed antimicrobial activity cannot be distinguished from the present data.

Zambrano et al. [41] reported antimicrobial activity of oven-dried and lyophilized Othello grape extracts against *Bacillus cereus* and *Bacillus subtilis*, with an MIC of 12.5 mg/mL. FDE also exhibited activity against these *Bacillus* spp. in the present study (MIC = 2.5–5 mg/mL); however, these values should not be interpreted as evidence of greater antimicrobial potency because differences in starting material, extract preparation, composition, and experimental conditions preclude direct quantitative comparison between the studies. The relatively weak antifungal activity of the investigated samples is consistent with previous findings [2,42,43] and may reflect the greater tolerance of fungi to wine-derived bioactive constituents, potentially associated with their complex cell wall structure and efficient stress-response and detoxification mechanisms.

OW and PDW exhibited no detectable antibiofilm activity within the tested concentration range. In contrast, measurable MBIC values were obtained for FDE. However, after normalization to starting-wine-volume equivalents, the lower numerical mass-based MBIC values of FDE should not be interpreted as evidence of stronger antibiofilm activity than OW or PDW. The results obtained in the present study are generally consistent with previously reported findings, although some differences were observed. Zambrano et al. [41] reported that *S. aureus* biofilms were particularly sensitive to Othello grape extracts, while Čomić et al. [2] demonstrated a strong inhibitory effect of dealcoholized red wine samples against *S. aureus* ATCC 6538 biofilms. In contrast, in the present study, OW and PDW did not exhibit detectable antibiofilm activity within the tested concentration range, whereas measurable effects were observed for FDE, particularly against *Bacillus cereus*. These discrepancies may be related to differences in phenolic concentration, sample preparation procedures, grape composition and experimental conditions.

The comparison between incubation periods also revealed a time-dependent difference in FDE activity, particularly against *B. cereus*, for which the MBIC increased from 2.5 mg/mL after 24 h to 10 mg/mL after 48 h. This observation may be related to the greater tolerance generally associated with more established biofilms, which possess a more complex architecture and a well-developed extracellular polymeric matrix that can limit the penetration and efficacy of antimicrobial agents [44,45].

The measurable antibiofilm activity of FDE may be related to its phenolic composition and matrix characteristics. Several phenolic compounds detected in the investigated samples, including quercetin, apigenin, gallic acid, and rutin, have previously been associated with antibiofilm activity [14]. These compounds interfere with key processes involved in biofilm development, such as bacterial adhesion, quorum sensing, and extracellular matrix production. Ethanol has also been reported to modulate biofilm formation in certain bacterial species, including *S. aureus*, under specific conditions [46], although its contribution under the conditions of the present study remains uncertain. The distinct activity profile of FDE may be associated with differences in its chemical composition and matrix characteristics; however, the present study does not allow the contribution of the evaporation procedure itself to be isolated.

The obtained results indicate that all tested wine samples exhibited protective effects against oxidative DNA damage induced by both hydroxyl and peroxyl radicals under the experimental conditions used. The observed preservation of DNA band integrity and fluorescence intensity indicates that the samples reduced oxidative DNA degradation in both experimental systems. In the Fenton reaction system, which generates highly reactive hydroxyl radicals, the positive control showed extensive DNA degradation. In the presence of wine samples, DNA integrity was better preserved, consistent with possible radical-scavenging and/or metal-chelating activity that may reduce oxidative damage [47]. Similarly, in the AAPH-induced peroxyl radical system, all samples attenuated DNA damage relative to the positive control [48]. These findings are consistent with previous reports describing the antioxidant properties of wine polyphenols, including their potential to scavenge free radicals and chelate transition metals [49,50].

The DNA-protective activity observed in OW, PDW, and FDE may be related, among other factors, to their phenolic composition, including the presence of epicatechin detected in all samples. Epicatechin is a flavan-3-ol with established antioxidant properties and has been reported to scavenge reactive oxygen species and limit oxidative damage [51,52]. Its presence may therefore contribute to the protective effects observed in the present study. However, because the samples were evaluated as complex mixtures and the contribution of individual compounds was not experimentally tested, the observed DNA-protective effects cannot be attributed exclusively to epicatechin or other phenolic constituents. Furthermore, the possible contributions of other sample components and matrix-related effects were not specifically assessed. In addition, the doses of OW and PDW were expressed as weighed amounts of liquid wine (approximately 3.1–50 nL), whereas those of FDE were expressed as mass of dry extract; because the density and dry-matter content of PDW were not determined, the doses could not be expressed on a common dry-matter or phenolic basis, and the DNA-protective effects should therefore be regarded as qualitative, without quantitative comparison between the liquid samples and FDE. Moreover, the samples were dried under ambient conditions for 24 h before the assay, during which oxidative changes in phenolic constituents cannot be excluded.

Taken together, the spectrophotometric, chromatographic, and biological analyses revealed distinct chemical and biological characteristics among the three investigated sample types. PDW exhibited a phenolic profile and biological activity pattern broadly comparable to those of OW, whereas FDE showed a distinct phenolic composition and biological activity profile while also showing DNA-protective activity. Given the comparative design of the present study, these differences should be interpreted as characteristics of the investigated samples rather than as definitive causal effects of the corresponding processing operations. Some limitations of the present study should also be noted. The individual contributions of volume reduction, thermal exposure, and ethanol and SO_2_ reduction could not be separated, as no evaporation control was included and titratable acidity, residual sugar, density, and dry matter were not determined. Phenolic compounds were identified by retention time, DAD spectra, and co-injection with authentic standards rather than by LC–MS; HPLC validation parameters (LOD/LOQ, recovery, repeatability) were not determined, and individual anthocyanins could not be quantified because authentic standards were unavailable. Finally, only one red wine was investigated, so the results cannot be extended to other wines, including white and rosé wines, which differ substantially in phenolic composition.

## 4. Materials and Methods

### 4.1. Vineyard and Harvest

The grapes used for the production of experimental wine were obtained from a private vineyard owned by the Milošević family, located in the Smederevska Palanka region (Serbia), at an altitude of approximately 150–200 m above sea level with southeastern exposure. The vineyard was cultivated under conventional viticultural practices. The wine was produced from Othello [*Vitis vinifera* × (*Vitis labrusca* × *Vitis riparia*)], an old red direct-producer hybrid grape cultivar. Vineyard protection included treatment with copper sulfate (CuSO_4_) in May, applied in accordance with the legally prescribed pre-harvest interval. Therefore, the presence of residual copper originating from vineyard treatment was not expected in the harvested grapes, minimizing the risk of direct contamination of the wine. Harvesting was performed manually during the period from September to October 2024. Grape clusters were collected intact, including stems, without prior separation of individual berries.

Following harvest, the grapes were crushed and subjected to spontaneous fermentation in the presence of pomace (skins, stems, and seeds), which lasted between 20 and 30 days. No commercial starter cultures (yeasts or lactic acid bacteria), sulfur dioxide, or other oenological additives were applied, allowing fermentation to proceed under natural microbiota. During fermentation, continuous contact between the must and solid grape components facilitated the extraction of phenolic compounds and color. Fermentation temperature was not actively controlled but followed natural fluctuations of the cellar environment. To support fermentation activity, a small amount of sugar (~5–10 g/L) was added. Upon completion of primary fermentation, the wine was subjected to two successive rackings and decantations to remove lees. The clarified wine was subsequently transferred to glass containers and stored for further maturation. After maturation, the wine contained 12% alcohol, and the pH was 3.54. The concentrations of total and free sulfur dioxide (SO_2_) were 5.2 mg/L and 2.1 mg/L, respectively, as determined by aeration–oxidation method according to OIV-MA-AS323-04A1 [53] and OIV-MA-AS323-04A2 [54]. After procurement, wine samples were stored at 5 °C until further experimental analysis.

#### 4.1.1. Preparation of Samples for Analysis

Three types of samples were investigated: (i) original wine (OW); (ii) processed dealcoholized wine (PDW), obtained by gentle rotary evaporation while maintaining the sample in liquid form; and (iii) fully evaporated dry wine extract (FDE), obtained by evaporation of the wine until no visible liquid phase remained and an apparently dry residue was obtained.

PDW was prepared by subjecting 500 mL of the original wine to rotary evaporation at 30 °C for 40 min. The treatment resulted in a final liquid volume of approximately 100 mL, and the sample was not subsequently adjusted to a predefined final volume. A visible precipitate formed on the walls of the flask during evaporation; it remained in the flask and was not included in the analyzed PDW sample. OW was not subjected to evaporation and was analyzed as received. Residual ethanol and free and total SO_2_ contents in OW and PDW were subsequently determined as described below. After processing, PDW contained 0.2% (*v*/*v*) ethanol, free and total SO_2_ below 1 mg/L, and had a pH of 3.39, compared with 12.0% (*v*/*v*) ethanol, 2.1 mg/L free and 5.2 mg/L total SO_2_, and pH 3.54 in OW (Section 4.1). These values were determined in PDW prepared again during revision from the same wine batch under identical processing conditions (Section 4.1.2).

FDE was prepared by subjecting 500 mL of the original wine to rotary evaporation at 40–45 °C for approximately 80 min, corresponding to the time required under the applied conditions for the visible liquid phase to disappear and an apparently dry residue to be obtained. Following evaporation, 13.585 g of apparently dry residue was recovered, corresponding to an extract yield of 27.17 g/L of starting wine (2.717% *w*/*v*). Residual moisture was not independently determined; therefore, the term “dry” is used operationally to describe the absence of a visible liquid phase rather than analytically confirmed complete dryness. The pH of FDE, measured after reconstitution at 30 mg/mL, was 3.52. The experimentally determined yield was used, where appropriate, to convert FDE data expressed on a dry-mass basis (mg/g) to wine-volume-equivalent values (mg/L of starting wine). Mass balances (recovery, % of OW) were calculated as the amount of each parameter in PDW (concentration × final volume) or in FDE (content per g × total extract mass) divided by the amount present in the 500 mL of OW used for their preparation, multiplied by 100. PDW and FDE were each prepared once from the same original wine batch. Subsequent measurements were performed in triplicate, unless otherwise stated. These replicate measurements were obtained from the same prepared sample and therefore do not represent independent technological replicates of the PDW or FDE preparation procedures.

#### 4.1.2. Physicochemical Characterization of the Samples

The pH values of OW and PDW were measured directly using a pH meter (C931, Consort, Turnhout, Belgium). Since FDE was obtained as a dry extract, its pH was determined after reconstitution by dissolving 0.3 g of FDE in 10 mL of distilled water (30 mg/mL). Ethanol content in OW and PDW was determined by ebulliometry using an ebulliometer (Ref. 160350D, Laboratoires Dujardin-Salleron, Noizay, France). The boiling temperatures of the reference standard and wine samples were recorded, and the corresponding alcohol contents were determined using the calibrated ebulliometer disc and expressed as % (*v*/*v*). Free and total sulfur dioxide (SO_2_) contents in OW and PDW were determined by the aeration–oxidation method according to OIV-MA-AS323-04A1 and OIV-MA-AS323-04A2 [53,54]. Sulfur dioxide released from the acidified sample was entrained by a gas stream and oxidised in a neutral hydrogen peroxide solution, and the resulting sulfuric acid was titrated with 0.01 M NaOH. The results were expressed as mg/L; concentrations below 1 mg/L, the reporting resolution of the method, were considered not detected. Ethanol and SO_2_ were not determined in FDE.

### 4.2. Investigation of Phenolic Composition of Wine Samples

#### 4.2.1. Determination of Total Phenolic Content (TPC)

The TPC was determined using the spectrophotometric method [55]. Prior to analysis, OW and PDW were diluted with methanol, whereas FDE was dissolved in methanol to obtain a stock solution of 30 mg/mL, which was further diluted with methanol (Zorka Pharma, Šabac, Serbia) to 1 mg/mL prior to analysis. For the reaction mixture, 2.5 mL of 10% Folin–Ciocalteu reagent (Sigma-Aldrich, St. Louis, MO, USA) diluted in water and 2.5 mL of 7.5% NaHCO_3_ (Sigma-Aldrich, St. Louis, MO, USA) were mixed with 0.5 mL of the prepared FDE, PDW, or OW sample. The samples were further incubated at 45 °C for 15 min. The absorbance was measured at λmax = 765 nm (spectrophotometer Jenway UV/VIS 6105 with the resolution range 1 nm). Measurements were performed in triplicate, and the results are expressed as mean ± SD. Pure methanol was used as the corresponding solvent blank. The same procedure was repeated for the gallic acid (Sigma-Aldrich, St. Louis, MO, USA), and the calibration curve was constructed. The TPC was expressed as gallic acid equivalent (mg of GAE/g of FDE or mg/L of PDW and OW, respectively).

#### 4.2.2. Determination of Total Flavonoid Content (TFC)

The FDE sample contained 1 mL of methanol solution of the extract in the concentration of 1 mg/mL (prepared by dilution of the 30 mg/mL methanolic stock solution) and 1 mL of 2% AlCl_3_ (Sigma-Aldrich, St. Louis, MO, USA) solution dissolved in methanol. The second group of samples contained 1 mL of PDW or OW and 1 mL of 2% AlCl_3_ solution dissolved in the methanol [56]. The absorbance was measured with the spectrophotometer Jenway UV/VIS 6105 with the resolution range of 1 nm. Measurements were performed in triplicate, and the results are expressed as mean ± SD. The same procedure was repeated for the rutin (Sigma-Aldrich, St. Louis, MO, USA), and the calibration line was constructed. Concentration TFC was expressed in terms of rutin equivalent (mg of RUE/g of FDE or mg of RUE/L of PDW and OW, respectively).

#### 4.2.3. Determination of Total Tannins Content (TTC)

The total tannin content (TTC) was determined spectrophotometrically according to the method described in [57], with slight modifications. For each sample, two aliquots containing 2 mL of FDE solution (1 mg/mL, prepared by dilution of the 30 mg/mL stock solution), PDW, or OW were mixed with 3 mL of concentrated HCl (Zorka Pharma, Šabac, Serbia) and 1 mL of distilled water. The first aliquot was incubated at 100 °C for 30 min, whereas the second aliquot served as the corresponding control and was supplemented with 0.5 mL of ethanol (Zorka Pharma, Šabac, Serbia). Absorbance was measured at 470, 520, and 570 nm, and the differences between the corresponding treated and control samples (ΔA470, ΔA520, and ΔA570) were calculated. The absorbance differences were converted to the corresponding ΔA520 values using the relationships ΔA520 = 1.1 × ΔA470 and ΔA520 = 1.54 × ΔA570, and the minimum ΔA520 value was used for TTC calculation. TTC was calculated according to the equation TTC = 15.7 × minimum (ΔA520) and expressed as mg/g for FDE and mg/L for OW and PDW. Measurements were performed in triplicate, and the results are presented as mean ± standard deviation (SD).

#### 4.2.4. Determination of Total Anthocyanin Content (TAC)

The total anthocyanin content (TAC) was determined spectrophotometrically according to the method described in [57], with slight modifications. For each sample, two aliquots containing 0.5 mL of FDE solution (30 mg/mL), PDW, or OW were mixed with 0.5 mL of 0.1% HCl in ethanol and 10 mL of 2% aqueous HCl. Subsequently, 4.4 mL of distilled water was added to one aliquot, while 4.4 mL of 13% sodium bisulfite (Sigma-Aldrich, St. Louis, MO, USA) solution was added to the second aliquot. The resulting mixtures were diluted 1:1, and absorbance was measured at 520 nm. A blank solution containing 4.9 mL of distilled water, 0.5 mL of 0.1% HCl in ethanol, and 10 mL of 2% aqueous HCl was used. The difference in absorbance (ΔA) between the corresponding samples was calculated, and TAC was determined by multiplying ΔA by 875. TAC was expressed as mg/g for FDE and mg/L for OW and PDW. For FDE, the TAC determined in the 30 mg/mL solution was divided by the solution concentration to obtain mg/g of dry extract. Measurements were performed in triplicate, and the results are presented as mean ± standard deviation (SD).

#### 4.2.5. HPLC Analysis of Polyphenolic Compounds

The chemicals used in this study, including the reference standards gallic acid, 3,4-dihydroxybenzoic acid, 3,5-dihydroxybenzoic acid, chlorogenic acid, caffeic acid, syringic acid, epicatechin, chrysin, p-coumaric acid, ferulic acid, sinapic acid, rutin, naringin, myricetin, quercetin, naringenin, and apigenin, as well as methanol, were obtained from Sigma-Aldrich (Steinheim, Germany). Acetonitrile and formic acid (HPLC grade) were obtained from Merck (Darmstadt, Germany). Individual standard stock solutions were prepared in methanol at a concentration of 1 mg/mL, stored under refrigerated conditions, and used within one week. For HPLC analysis, FDE was dissolved in methanol at a concentration of 1 mg/mL, whereas OW and PDW were diluted prior to injection, and the measured concentrations were corrected for the corresponding dilution factors.

The identification and quantification of individual phenolic compounds were performed using a Shimadzu Prominence HPLC system (Shimadzu, Kyoto, Japan) equipped with an SPD-M20A photodiode array (PDA) detector. Chromatographic separation was achieved on a Hypersil Gold aQ C18 column (150 × 4.6 mm, 5 µm) maintained at ambient temperature. The mobile phase consisted of (A) 0.1% formic acid in deionized water and (B) 0.1% formic acid in acetonitrile. Gradient elution started at 2% B, which was maintained for the first 2 min, followed by a gradual increase to 95% B over 45 min. The flow rate was 1.0 mL/min and the injection volume was 20 µL.

PDA chromatograms were recorded at 254, 280, 360, and 520 nm to monitor different classes of phenolic compounds. Data acquisition and processing were performed using Shimadzu LabSolutions software (version 5.93). Phenolic compounds were identified by comparison of their retention times and DAD UV spectra with those of authentic reference standards. Compound identity was additionally verified by co-injection with the corresponding reference standards. The retention times (tRs), wavelengths used for quantification, and wavelengths of maximum UV absorption (λmax) for the individual compounds are provided in Supplementary Table S1. Representative HPLC-DAD chromatograms of OW, PDW, and FDE recorded at 254, 280, 360, and 520 nm, with selected identified peaks and their corresponding retention times, are provided in Supplementary Figures S1–S4. Quantification was performed by external calibration using the corresponding reference standards.

### 4.3. Investigation of Antioxidant Activity by DPPH and ABTS Assays

The antioxidant activity was evaluated using the DPPH and ABTS assays as described in Mavric-Scholze et al. [58] and Avdović et al. [6,7], with slight modifications. Wine samples were prepared at various concentrations by dilution with methanol. Specifically, OW and PDW samples were prepared in the concentration range of 1–2 µL/mL, while the FDE sample was prepared in the range of 0.1–0.5 mg/mL. For the DPPH assay, a 1,1-diphenyl-2-picrylhydrazyl (DPPH, Merck, KGaA, Darmstadt, Germany) solution (50 µM) was prepared in methanol. Equal volumes of the sample and DPPH solution were mixed and incubated in the dark for 20 min. The absorbance was then measured at 517 nm.

For the ABTS assay, the ABTS, radical cation (ABTS^•+^) was generated by reacting an 2,2′-azino-bis(3-ethylbenzothiazoline-6-sulfonic acid (ABTS, Merck, KGaA, Darmstadt, Germany) stock solution (7 mM) with potassium persulfate (2.45 mM) and allowing the mixture to stand in the dark at room temperature for 12–16 h before use. The ABTS^•+^ solution was then diluted with methanol to obtain an absorbance of 0.70 at 734 nm. An appropriate volume of the sample was mixed with the ABTS^•+^ solution and the absorbances were measured at 734 nm.

Antioxidant activity was reported as Trolox equivalents (mmol TE/L). Calibration curves were constructed using standard Trolox solutions in the range of 2–8 µmol/L.

### 4.4. Investigation of Antimicrobial Activity of Wine Samples

#### 4.4.1. Test Microorganisms

The antimicrobial activity of the tested samples (FDE, PDW and OW) was evaluated against a panel of bacterial and fungal strains. The list of tested microorganisms is presented in Table 4. Clinical isolations were kindly provided by the Institute of Public Health, Kragujevac, while reference (ATCC) strains and fungal species were obtained from the culture collection of the Microbiology Laboratory, Faculty of Science, University of Kragujevac.

#### 4.4.2. Preparation of Microbial Suspensions

Bacterial and yeast suspensions were prepared using the direct colony method [59]. The turbidity of the initial suspensions was adjusted to 0.5 McFarland standard using a densitometer (BioSan, Riga, Latvia), corresponding to approximately 10^8^ CFU/mL for bacteria and 10^6^ CFU/mL for yeasts. Subsequently, 1:100 dilutions of the initial bacterial and yeast suspensions were prepared in sterile 0.85% saline. Fungal spore suspensions were prepared by gentle scraping of spores from agar slopes with actively growing mycelia, followed by dilution (1:1000) in sterile 0.85% saline.

#### 4.4.3. Microdilution Assay

The antimicrobial activity of the tested samples was assessed using the broth microdilution method with resazurin as an indicator of microbial growth [60]. Twofold serial dilutions of OW and PDW were prepared in sterile 96-well microtiter plates to obtain final concentrations up to 25 µL/mL, while the extract was tested in concentrations up to 5 mg/mL. Mueller–Hinton broth (Torlak, Belgrade, Serbia) was used for bacteria and Sabouraud dextrose broth (Torlak, Belgrade, Serbia) for yeasts and fungi, with a final volume of 0.1 mL per well.

Microtiter plates were inoculated with microbial suspensions to achieve a final concentration of 5 × 10^5^ CFU/mL for bacteria and 5 × 10^3^ CFU/mL for yeasts and fungi. After inoculation, resazurin solution (Alfa Aesar GmbH & Co., Karlsruhe, Germany) was added to each well as a growth indicator. The plates were incubated at 37 °C for 24 h for bacteria, at 28 °C for 48 h for yeasts, and at 28 °C for 72 h for filamentous fungi. The minimum inhibitory concentration (MIC) was defined as the lowest concentration of the tested sample that prevented the color change in resazurin from blue to pink, indicating inhibition of microbial growth. For filamentous fungi, MIC was defined as the lowest concentration that inhibited visible mycelial growth. To determine the minimum microbiocidal concentration (MMC), aliquots (10 μL) from wells without visible growth were inoculated onto appropriate agar media and incubated under the same conditions. The lowest concentration yielding no colony formation was defined as the MMC.

Tetracycline (Sigma Chemicals Co., St. Louis, MO, USA) was used as the antibacterial reference compound and was dissolved in Mueller–Hinton broth. Amphotericin B (Sigma Chemicals Co., USA) was used as the antifungal reference compound and was dissolved in Sabouraud dextrose broth. FDE was dissolved in dimethyl sulfoxide (DMSO, Acros Organics, Thermo Fisher Scientific, Geel, Belgium) and subsequently diluted with culture medium to obtain the required test concentrations. The final DMSO concentration in the microtitration wells was 0.5% (*v*/*v*). A corresponding solvent control containing 0.5% DMSO was included to verify that the solvent itself did not affect microbial growth. Each experiment included sterility and growth controls. Experiments were performed in duplicate, and reproducible MIC and MMC values are reported.

### 4.5. Biofilm Formation and Antibiofilm Assay

The biofilm-forming ability of *Staphylococcus aureus* ATCC 25923, *S. aureus*, *Bacillus subtilis* ATCC 6633, *B. subtilis* IP 5832, and *B. cereus* was evaluated using a modified crystal violet microtiter plate assay, according to the method described by Stepanović et al. [61]. Briefly, 100 μL of Tryptic Soy Broth (Torlak, Belgrade, Serbia) was dispensed into each well of sterile 96-well flat-bottom polystyrene tissue culture-treated microplates. Subsequently, 10 μL of bacterial suspension adjusted to the 0.5 McFarland standard was added to each well. The plates were incubated at 37 °C for 24 and 48 h under static conditions.

Following incubation, planktonic cells were carefully removed, and the wells were gently washed with sterile distilled water to eliminate non-adherent bacteria. The remaining biofilms were fixed by exposure to hot air at 55 °C for 20 min. Thereafter, the adherent biomass was stained with 0.1% (*w*/*v*) crystal violet (Fisher Scientific, Geel, Belgium) for 20 min at room temperature. Excess stains were removed by washing the wells twice with distilled water, and the plates were air-dried in an inverted position. The retained dye was subsequently solubilized with 100 μL of 33% (*v*/*v*) acetic acid (Betahem d.o.o., Belgrade, Serbia), followed by gentle pipetting to ensure complete dissolution. Biofilm biomass was quantified by measuring the optical density (OD) at 550 nm using a microplate reader (RT-2100C, Rayto, Shenzhen, China).

The antibiofilm activity of the tested wine samples was determined using the same assay with minor modifications. Twofold serial dilutions of the samples were prepared directly in the wells to obtain final concentrations ranging from 25 to 0.195 µL/mL for OW and PDW, while the FDE was tested in the range of 10 to 0.078 mg/mL. After the addition of 10 μL of bacterial suspension (0.5 McFarland standard), the plates were incubated at 37 °C for 24 and 48 h.

Subsequent steps, including removal of planktonic cells, washing, fixation, staining, and quantification, were performed as described above. The minimum biofilm inhibitory concentration (MBIC) was defined as the lowest concentration of the tested sample that prevented visible biofilm formation, as determined by optical density measurements at 550 nm. Tetracycline was included as a positive control, and its MBIC was determined after 48 h of incubation under the same assay conditions and expressed in µg/mL. Controls included broth or broth with different wine samples to check sterility and nonspecific binding. To compensate for background absorbance, OD readings from sterile medium, extracts, fixative, and dye were averaged and subtracted from all test values. All tests were performed in duplicate.

### 4.6. In Vitro DNA-Protective Capacity

#### 4.6.1. Hydroxyl Radical-Induced DNA Damage

The protective potential of wine samples against hydroxyl radical-mediated DNA damage was assessed using salmon sperm DNA (Carl Roth, GmbH, Karlsruhe, Germany), following a modified protocol previously described by Lin et al. [62]. For sample preparation, 1 mg of each liquid wine sample (OW and PDW) was weighed into an Eppendorf tube and mixed with 1 mL of methanol; assuming a density close to 1 g/mL, this corresponds to approximately 1 µL of wine. For FDE, 1 mg of the dry extract was weighed and dissolved in 1 mL of methanol. Aliquots corresponding to 3.125, 6.25, 12.5, 25, and 50 μg of each sample were transferred into separate Eppendorf tubes. For OW and PDW, these amounts correspond to approximately 3.1–50 nL of wine, and for FDE to 3.125–50 μg of dry extract. Based on the TPC values (Table 1), these doses corresponded to approximately 2.4–38.7 ng GAE for OW, 2.6–41.6 ng GAE for PDW, and 0.12–1.98 μg GAE for FDE. Because the density and dry-matter content of PDW were not determined, the doses of the liquid samples could not be expressed as amounts of wine solids; the doses of the liquid wine samples and of FDE are therefore not directly comparable on a quantitative basis, and the DNA-protective effects are interpreted qualitatively for each sample. The methanol was allowed to evaporate from the open tubes for 24 h at room temperature under ambient atmospheric conditions. Subsequently, 45 μL of sodium phosphate buffer (Sigma-Aldrich, St. Louis, MO, USA) (0.2 mol/L, pH 7.4), 10 μL of salmon sperm DNA (Carl Roth, GmbH, Karlsruhe, Germany) solution (10 mg/mL in 0.9% NaCl), 0.9 μL of FeSO_4_ (Merck KGaA, Darmstadt, Germany) (180 μmol/L), and 3.6 μL of H_2_O_2_ (Sigma-Aldrich (St. Louis, MO, USA) (600 μmol/L) were added to each tube. The reaction mixtures were incubated at room temperature for 15 min, after which the reaction was terminated by the addition of 10 μL EDTA (Sigma-Aldrich, St. Louis, MO, USA) solution (1 μmol/L). Salmon sperm DNA solution served as the negative control, while quercetin (Merck KGaA, Darmstadt, Germany) (50 μg/mL) was used as the reference antioxidant [63].

Following incubation, 10 μL of each sample was applied onto a 1% agarose gel prepared in 1× TAE buffer containing ethidium bromide (Alfatrade Enterprise D.O.O. (Serva Electrophoresis GmbH, Heidelberg, Germany) (10 mg/mL). Electrophoresis was carried out at 100 V using a BlueMarine™ 100 electrophoresis unit (Serva Electrophoresis GmbH, Heidelberg, Germany). DNA bands were visualized under UV light at 365 nm using a UV transilluminator (Vilber Lourmat, Collégien, France), photographed, and the band intensities were quantified using ImageJ software version 1.48 for Windows (Softonic International, Barcelona, Spain).

#### 4.6.2. Peroxyl Radical-Induced DNA Damage

The ability of wine samples (3.125, 6.25, 12.5, 25, and 50 μg of each sample; see Section 4.6.1) to protect DNA against peroxyl radical-induced oxidative damage was evaluated according to the method reported by Zhang et al. [64]. Sample preparation was performed as described in Section 4.6.1. Briefly, the methanolic sample solutions were prepared as described in Section 4.6.1, and aliquots corresponding to 3.125, 6.25, 12.5, 25, and 50 μg of each sample were transferred into separate open Eppendorf tubes, and the methanol was allowed to evaporate for 24 h at room temperature under ambient atmospheric conditions. Subsequently, 10 μL of salmon sperm DNA and 45 μL of phosphate buffer were added to each tube. Oxidative stress was initiated by adding 10 μL of freshly prepared 200 mM 2,2′-azobis(2-methylpropionamidine) dihydrochloride (AAPH, Acros, Organics, Newark, NJ, USA). The reaction mixtures were then incubated at 37 °C for 4 h following vortexing. Subsequently, DNA samples were separated by electrophoresis on 1% agarose gel supplemented with ethidium bromide (10 mg/mL). The resulting DNA bands were visualized under UV illumination at 365 nm using a UV transilluminator (Vilber Lourmat, France), photographed, and quantified using ImageJ software.

### 4.7. Statistical Analysis

For the physicochemical, phenolic, HPLC, and antioxidant measurements, replicate determinations were performed on the same prepared samples and the results are presented as mean ± SD. Because these replicates did not represent independently prepared wine samples or independent technological replicates, they were not treated as independent observations for inferential comparisons among OW, PDW, and FDE. For the DNA protection assays, statistical analysis was performed using one-way analysis of variance (ANOVA). Normality of data distribution and homogeneity of variances were assessed using the Kolmogorov–Smirnov and Levene’s tests, respectively. When homogeneity of variances was satisfied, Bonferroni post hoc comparisons were applied, whereas Dunnett’s T3 test was used when the assumption of homogeneity of variances was not met. Differences were considered statistically significant at *p* < 0.05. All statistical analyses were performed using SPSS Statistics version 13.0 for Windows.

## 5. Conclusions

The present comparative pilot study revealed distinct chemical and biological characteristics among OW, PDW, and FDE derived from the same red wine. PDW showed higher measured phenolic concentrations than OW and a broadly similar chromatographic profile; however, these higher concentrations reflected an approximately five-fold volume reduction, as PDW retained only approximately 22–30% of the phenolic constituents of the starting wine. FDE displayed a more distinct phenolic profile and retained approximately 68% of the anthocyanins of the starting wine. OW and PDW exhibited comparable antioxidant and antimicrobial activities, whereas only FDE showed measurable antibiofilm activity within the tested concentration ranges, and all samples exhibited in vitro protective activity against hydroxyl- and peroxyl radical-induced DNA damage. Because FDE was tested on a mass basis and the samples differed in matrix composition, its biological activities were not directly ranked against those of the liquid samples.

These observations are restricted to the specific samples investigated and should not be interpreted as definitive effects of dealcoholization or evaporation to dryness. Nevertheless, dealcoholized wine and dry wine extracts may represent potential starting materials for low-alcohol functional beverages or wine-derived food ingredients, provided that processing conditions limiting phenolic losses are developed and their biological relevance is confirmed.

Several limitations should be considered when interpreting these findings. The study was based on a single wine batch, while PDW and FDE were each prepared once without independent technological replicates or process controls. Therefore, the findings should be regarded as a comparative pilot assessment and cannot be generalized to other wines or used to attribute the observed differences to individual processing steps. Future studies should include multiple wines, independently replicated processing treatments, appropriate process controls, and more comprehensive physicochemical characterization to confirm the reproducibility and broader applicability of the observed patterns.

## Figures and Tables

**Table 1:** Total phenolic, flavonoid, tannin, and anthocyanin contents in the investigated wine samples.

Samples	TPC	TFC	TTC	TAC
OW (mg/L)	773.56 ± 7.05	72.84 ± 0.84	60.58 ± 0.30	28 ± 1
PDW (mg/L)	831.91 ± 9.82	82.49 ± 1.42	70.73 ± 0.34	42 ± 2
FDE (mg/g)	39.56 ± 0.62	7.85 ± 0.20	2.47 ± 0.01	0.70 ± 0.03
FDE wine equivalent (mg/L)	1074.85 ± 16.85	213.28 ± 5.43	67.11 ± 0.27	19.02 ± 0.91
PDW recovery (% of OW)	22	23	23	30
FDE recovery (% of OW)	139	293	111	68

Results are presented as the mean ± standard deviation (SD). OW, original wine; PDW, processed dealcoholized wine; FDE, fully evaporated dry wine extract; TPC, total phenolic content, expressed as mg gallic acid equivalents (GAE); TFC, total flavonoid content, expressed as mg rutin equivalents (RUE); TTC, total tannin content; TAC, total anthocyanin content. FDE values were originally determined on a dry-mass basis (mg/g) and were additionally converted to wine-volume equivalents (mg/L of starting wine) using the experimentally determined dry-extract yield of 27.17 g/L. Recovery (% of OW) was calculated as the amount of each parameter in PDW (concentration × final volume of approximately 100 mL) or in FDE (content per g × total extract mass of 13.585 g) relative to the amount present in the 500 mL of OW used for their preparation.

**Table 2:** Polyphenolic compound content in the investigated wine samples.

Compounds	Samples			
	OW (mg/L)	PDW (mg/L)	FDE (mg/g)	FDE Wine Equivalent (mg/L)
Gallic acid	34.09 ± 0.32	12.30 ± 0.45	0.79 ± 0.02	21.46 ± 0.54
3,4-DHB	4.54 ± 1.00	1.42 ± 0.60	0.84 ± 0.04	22.82 ± 1.09
3,5-DHB	4.03 ± 0.17	2.31 ± 0.03	0.16 ± 0.02	4.35 ± 0.54
Chlorogenic acid	20.44 ± 1.28	22.20 ± 0.09	1.28 ± 0.01	34.78 ± 0.27
Caffeic acid	10.97 ± 0.30	14.49 ± 0.08	0.29 ± 0.03	7.88 ± 0.82
Syringic acid	25.83 ± 0.44	11.51 ± 0.16	0.45 ± 0.02	12.23 ± 0.54
Epicatechin	52.38 ± 1.03	159.59 ± 0.20	5.10 ± 0.06	138.57 ± 1.63
p-Coumaric acid	9.00 ± 0.20	12.39 ± 1.69	0.34 ± 0.02	9.24 ± 0.54
Ferulic acid	12.88 ± 0.08	23.89 ± 0.12	0.38 ± 0.01	10.32 ± 0.27
Sinapic acid	33.27 ± 1.69	22.16 ± 0.12	1.17 ± 0.05	31.79 ± 1.36
Rutin	13.73 ± 0.93	28.19 ± 0.06	0.27 ± 0.01	7.34 ± 0.27
Naringin	39.06 ± 1.36	41.41 ± 0.62	0.63 ± 0.05	17.12 ± 1.36
Myricetin	18.86 ± 0.88	13.02 ± 0.34	0.18 ± 0.07	4.89 ± 1.90
Quercetin	0.55 ± 0.01	2.60 ± 0.11	0.08 ± 0.01	2.17 ± 0.27
Naringenin	25.29 ± 1.03	22.41 ± 0.15	0.46 ± 0.09	12.50 ± 2.45
Apigenin	2.28 ± 0.01	8.61 ± 0.64	0.56 ± 0.01	15.22 ± 0.27
Chrysin	0.41 ± 0.01	0.43 ± 0.02	0.08 ± 0.01	2.17 ± 0.27

Results are presented as mean value ± SD; OW—original wine; PDW—processed dealcoholized wine; FDE—fully evaporated dry wine extract; FDE concentrations were originally determined on a dry-mass basis (mg/g) and were additionally converted to wine-volume equivalents (mg/L of starting wine) using the experimentally determined dry-extract yield of 27.17 g/L.

**Table 3:** Antioxidant activity of the investigated samples.

Samples	Radical Scavenging Activity	
	Trolox Equivalent (mmol TE/L)	
	DPPH	ABTS
OW	0.98 ± 0.09	4.77 ± 0.15
PDW	1.17 ± 0.09	4.62 ± 0.54
FDE	9.33 ± 0.04	49.37 ± 0.27

Results are presented as mean value ± SD; OW—original wine; PDW—processed dealcoholized wine; FDE—fully evaporated dry wine extract.

**Table 4:** The antimicrobial activity of tested wine samples.

Wine Samples/Antibiotics/ Antimycotics	OW (µL/mL)		PDW (µL/mL)		FDE (mg/mL)		FDE Wine Equivalent (µL/mL)		Tetracycline (µg/mL)		Amphotericin B (µg/mL)	
Species	MIC	MMC	MIC	MMC	MIC	MMC	MIC	MMC	MIC	MMC	MIC	MMC
*Lactiplantibacillus plantarum*	>25	>25	>25	>25	5	5	184.03	184.03	0.16	1	/	/
*Bacillus subtilis* IP 5832	>25	>25	>25	>25	5	>5	184.03	>184.03	n.d.	n.d.	/	/
*Bacillus subtilis* ATCC 6633	25	25	25	25	2.5	5	92.01	184.03	0.25	0.37	/	/
*Bacillus cereus*	25	>25	25	>25	2.5	5	92.01	184.03	0.25	0.5	/	/
*Staphylococcus aureus*	0.391	6.25	<0.391	6.25	<0.156	5	<5.74	184.03	<0.06	<0.06	/	/
*Staphylococcus aureus* ATCC 25923	0.391	12.5	0.391	12.5	<0.156	0.625	<5.74	23.00	1.5	3	/	/
*Proteus mirabilis*	>25	>25	>25	>25	5	5	184.03	184.03	>128	>128	/	/
*Proteus mirabilis* ATCC 12453	>25	>25	>25	>25	5	>5	184.03	>184.03	15.62	31.25	/	/
*Pseudomonas aeruginosa*	25	>25	25	>25	5	>5	184.03	>184.03	>128	>128	/	/
*Pseudomonas aeruginosa* ATCC 27853	>25	>25	>25	>25	5	>5	184.03	>184.03	4	32	/	/
*Escherichia coli*	>25	>25	>25	>25	>5	>5	>184.03	>184.03	2	6	/	/
*Escherichia coli* ATCC 25922	>25	>25	>25	>25	>5	>5	>184.03	>184.03	4	6	/	/
*Candida albicans*	25	25	25	25	5	>5	184.03	>184.03	/	/	0.98	1.95
*Saccharomyces boulardii*	>25	>25	>25	>25	>5	>5	>184.03	>184.03	/	/	<0.098	<0.098
*Aspergillus flavus* ATCC 9170	>25	>25	>25	>25	>5	>5	>184.03	>184.03	/	/	0.98	15.62
*Aspergillus niger* ATCC 16404	>25	>25	>25	>25	>5	>5	>184.03	>184.03	/	/	0.195	0.39
*Penicillium italicum*	>25	>25	>25	>25	>5	>5	>184.03	>184.03	/	/	<0.098	1.56
*Penicillium chrysogenum*	>25	>25	>25	>25	>5	>5	>184.03	>184.03	/	/	0.195	0.39

OW—original wine; PDW—processed dealcoholized wine; FDE—fully evaporated dry wine extract; MIC—minimum inhibitory concentration; MMC—minimum microbiocidal concentration; n.d.—not determined; FDE wine-equivalent values were calculated from the experimentally determined dry-extract yield of 27.17 g/L of starting wine (27.17 mg FDE/mL wine).

**Table 5:** Antibiofilm activity of tested wine samples.

Samples	OW (µL/mL)		PDW (µL/mL)		FDE (mg/mL)		FDE Wine Equivalent (µL/mL)		Tetracycline (µg/mL)
Species	MBIC (24 h)	MBIC (48 h)	MBIC (24 h)	MBIC (48 h)	MBIC (24 h)	MBIC (48 h)	MBIC (24 h)	MBIC (48 h)	MBIC (48 h)
*S. aureus* ATCC 25923	>25	>25	>25	>25	10	10	368.05	368.05	250
*S. aureus*	>25	>25	>25	>25	10	10	368.05	368.05	˃500
*B. subtilis* ATCC 6633	>25	>25	>25	>25	10	>10	368.05	>368.05	<15.62
*B. cereus*	>25	>25	>25	>25	2.5	10	92.01	368.05	62.50

OW—original wine; PDW—processed dealcoholized wine; FDE—fully evaporated dry wine extract; MBIC—Minimum Biofilm Inhibitory Concentration; FDE values are presented both as the experimentally tested mass concentrations (mg/mL) and as starting-wine-volume equivalents (µL/mL). Wine-equivalent values were calculated using the experimentally determined dry-extract yield of 27.17 g/L of starting wine (27.17 mg FDE/mL wine). Tetracycline was used as a positive control, and its MBIC was determined after 48 h of incubation.

## Data Availability

Data are contained within the article.

## Supplementary Materials

The following supporting information can be downloaded at https://www.mdpi.com/article/10.3390/molecules31213843/s1, Figure S1: HPLC chromatograms of original wine (OW), processed dealcoholized wine (PDW) and fully evaporated dry wine extract (FDE) obtained at 254 nm; Figure S2: HPLC chromatograms of original wine (OW), processed dealcoholized wine (PDW) and fully evaporated dry wine extract (FDE) obtained at 280 nm; Figure S3: HPLC chromatograms of original wine (OW), processed dealcoholized wine (PDW) and fully evaporated dry wine extract (FDE) obtained at 360 nm; Figure S4: HPLC chromatograms of original wine (OW), processed dealcoholized wine (PDW) and fully evaporated dry wine extract (FDE) obtained at 520 nm; Table S1: Chromatographic and UV-DAD parameters used for the identification and quantification of phenolic compounds.

## Abbreviations

The following abbreviations are used in this manuscript:

TPC	Total phenolic contents
TFC	Total flavonoid contents
TTC	Total tannin contents
TAC	Total anthocyanin contents
OW	Original wine
PDW	Processed dealcoholized wine
FDE	Fully evaporated dry wine extract
DNA	Deoxyribonucleic acid
TPAC	Total proanthocyanidin content
EPS	Extracellular polymeric substance
SD	Standard deviation
DPPH	2,2-diphenyl-1-picrylhydrazyl
ABTS	2,2′-azino-bis(3-ethylbenzothiazoline-6-sulfonic acid
TE	Trolox equivalent
MIC	Minimum inhibitory concentrations
MMC	Minimum microbiocidal concentration
MBIC	Minimum Biofilm Inhibitory Concentration
H_2_O_2_	Hydrogen peroxide
AAPH	2,2′-azobis(2-amidinopropane) dihydrochloride
ROS	Reactive oxygen species
CuSO_4_	Copper sulfate
GAE	Gallic acid equivalent
RUE	Rutin equivalent
PDA	Photodiode array
HPLC	High-performance liquid chromatography
ATCC	American Type Culture Collection
CFU	Colony-forming units
DMSO	Dimethyl Sulfoxide
OD	Optical density
ANOVA	One-way analysis of variance
