# Supplementary material for: Comparative Phenolic Profiling and Biological Evaluation of Original Wine, Processed Dealcoholized Wine, and Fully Evaporated Dry Wine Extract
Source: Molecules. 2026 Oct 24;31(21):3843. doi: 10.3390/molecules31213843 (PMC13764942; doi:10.3390/molecules31213843)
Supplement: Supplementary file 1 [file molecules-31-03843-s001.zip › molecules-4597579-supplementary.pdf]

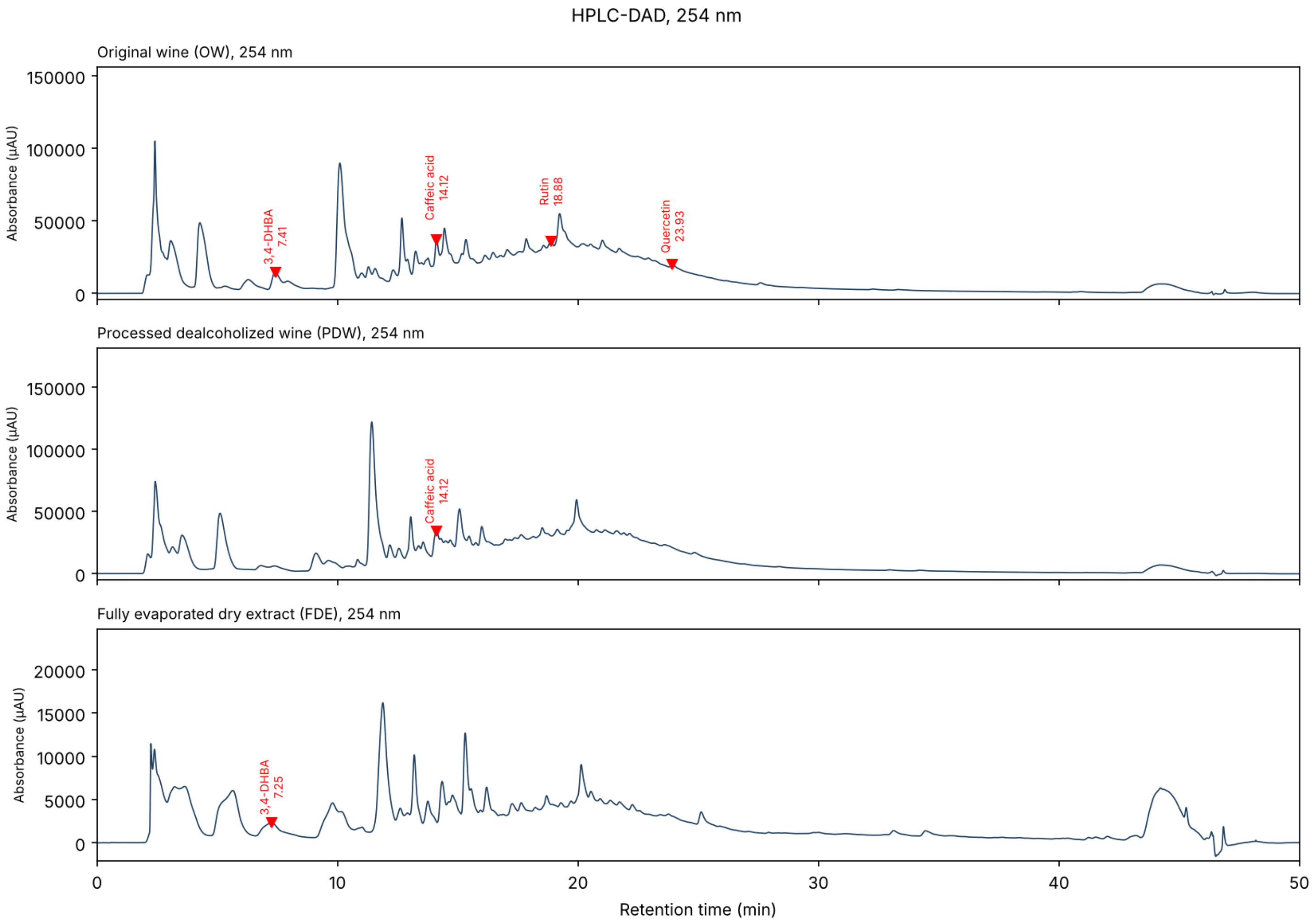

Figure S1. HPLC chromatograms of original wine (OW), processed dealcoholized wine (PDW) and fully evaporated dry wine extract (FDE) obtained at 254 nm

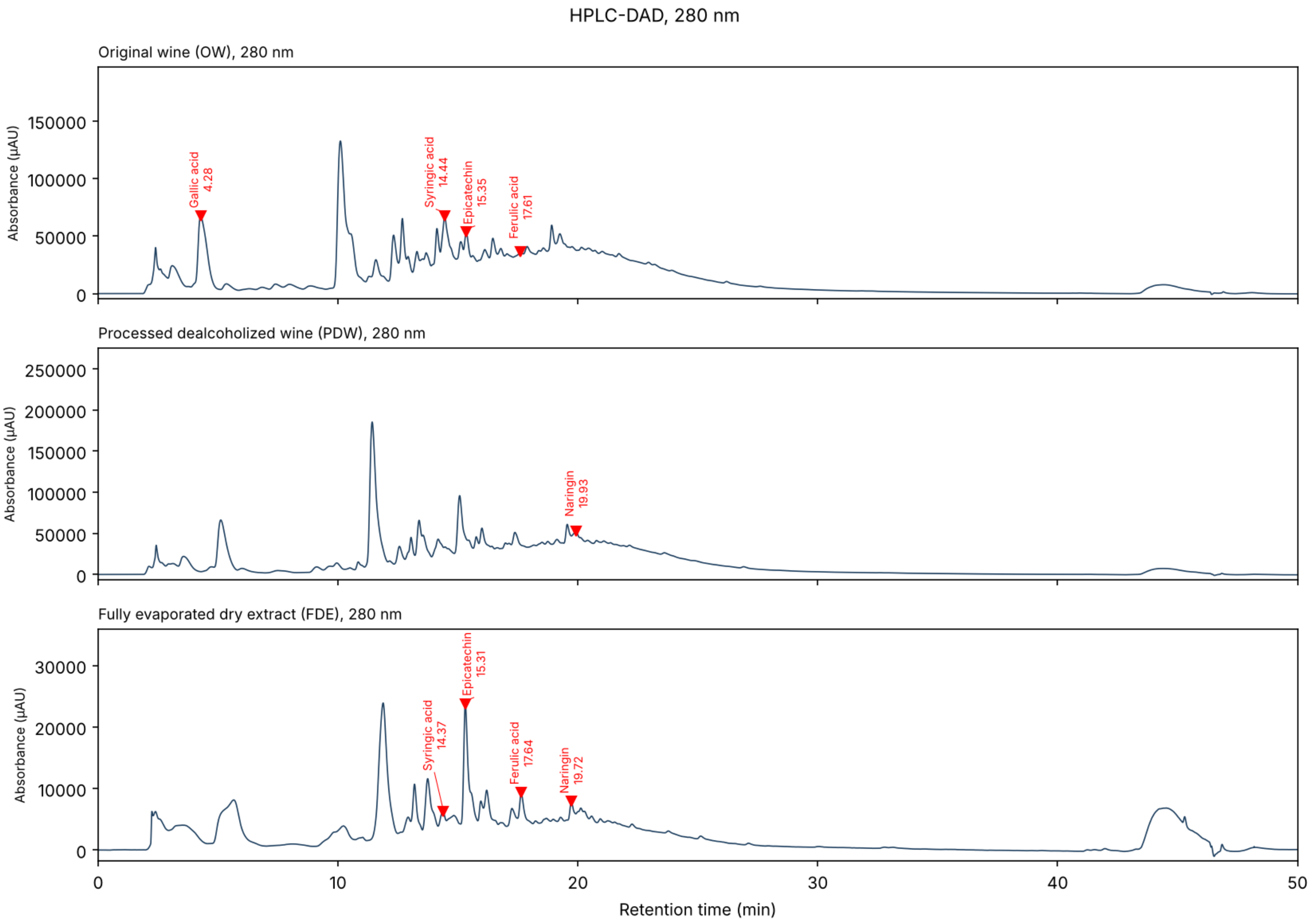

Figure S2. HPLC chromatograms of original wine (OW), processed dealcoholized wine (PDW) and fully evaporated dry wine extract (FDE) obtained at 280 nm

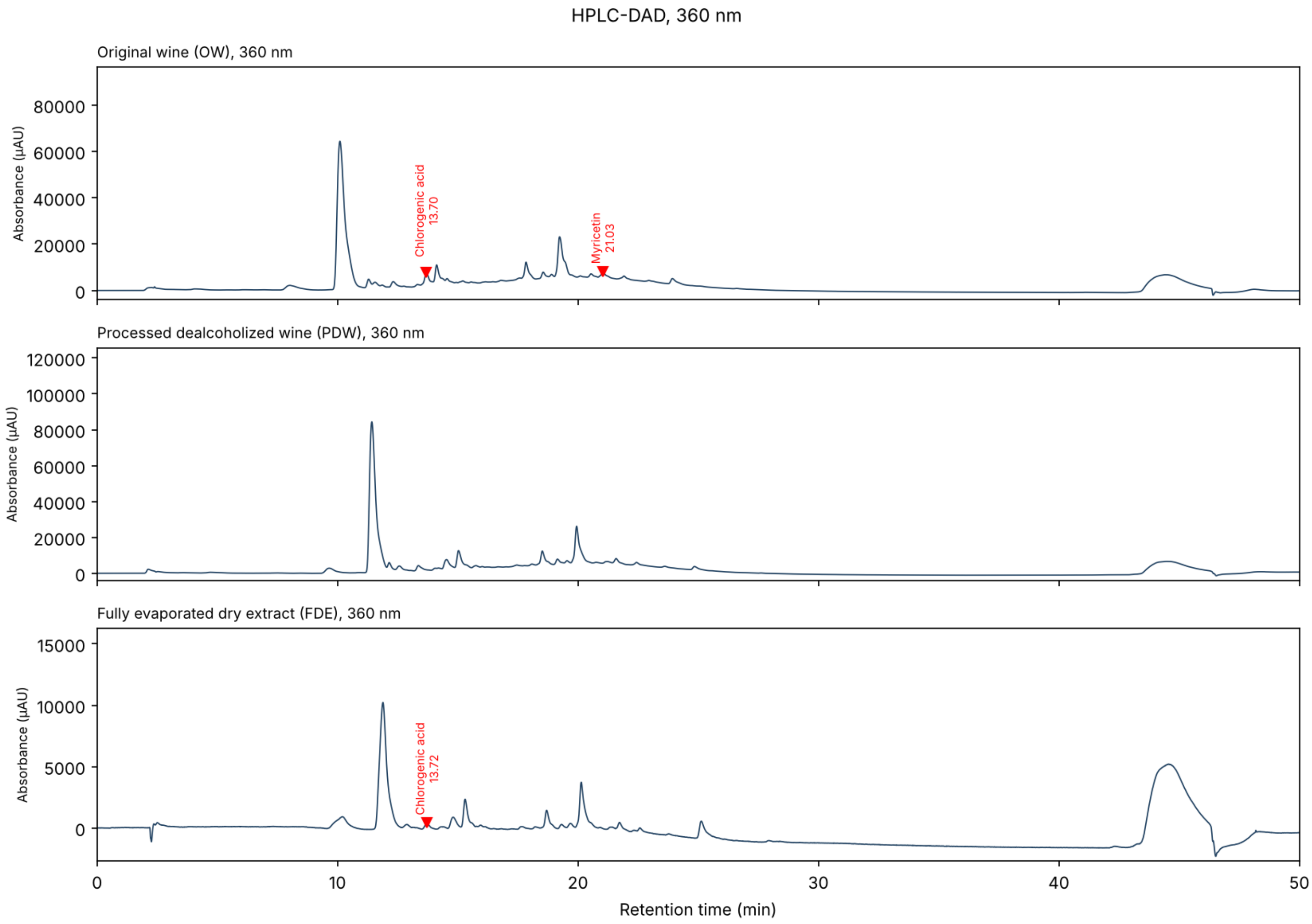

Figure S3. HPLC chromatograms of original wine (OW), processed dealcoholized wine (PDW) and fully evaporated dry wine extract (FDE) obtained at 360 nm

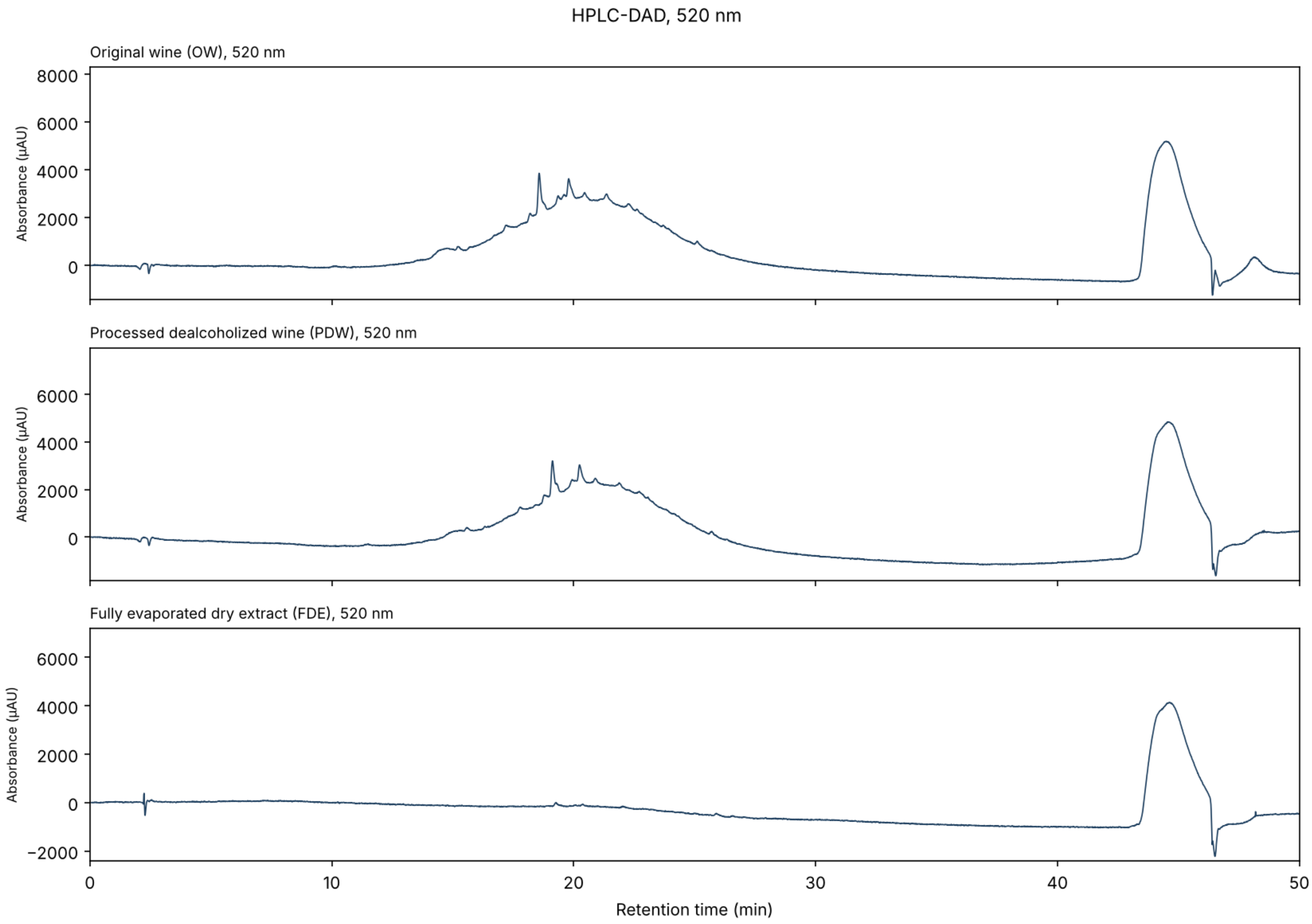

Figure S4. HPLC chromatograms of original wine (OW), processed dealcoholized wine (PDW) and fully evaporated dry wine extract (FDE) obtained at 520 nm

Supplementary Table S1. Chromatographic and UV-DAD parameters used for the identification and quantification of phenolic compounds.

Compound	tR (min)	Quantification wavelength (nm)	λmax (nm)
Gallic acid	4.333	280	271
3,4-DHBA	7.293	254	294
3,5-DHBA	8.015	254	250
Chlorogenic acid	13.790	360	330
Caffeic acid	14.094	254	322
Syringic acid	14.485	280	276
Epicatechin	15.328	280	278
p-Coumaric acid	16.451	360	310
Ferulic acid	17.639	280	320
Sinapic acid	18.040	254	318
Rutin	18.893	254	258
Naringin	19.860	280	284
Myricetin	21.112	360	376
Quercetin	24.023	254	258
Naringenin	24.857	280	288
Apigenin	26.530	280	270
Chrysin	31.468	280	272

tR, retention time; **λmax**, wavelength of maximum UV absorption.
